# Cyclin D1/D2–CDK4 Drives Cell Migration by Orchestrating Cytoskeletal Dynamics Through a TGFβ–FAK–Rac1 Axis

**DOI:** 10.3390/ijms27031228

**Published:** 2026-01-26

**Authors:** Ruifang Guo, Yihang Wang, Aiwen Zhang, Siwanon Jirawatnotai, Chen Chu, Lijun Liu

**Affiliations:** 1 Key Laboratory of Bioresource Research and Development of Liaoning Province, College of Life and Health Sciences, Northeastern University, Shenyang 110169, China; 2 National Frontiers Science Center for Industrial Intelligence and Systems Optimization, Northeastern University, Shenyang 110819, China; 3 Key Laboratory of Data Analytics and Optimization for Smart Industry (Northeastern University), Ministry of Education, Shenyang 110819, China; 4 Siriraj Center of Research Excellence for Precision Medicine and Systems Pharmacology, Faculty of Medicine Siriraj Hospital, Mahidol University, Bangkok 10700, Thailand; 5 Department of Pharmacology, Faculty of Medicine Siriraj Hospital, Mahidol University, Bangkok 10700, Thailand; 6 Department of Cancer Biology, Dana-Farber Cancer Institute, Boston, MA 02215, USA; 7 Department of Genetics, Blavatnik Institute, Harvard Medical School, Boston, MA 02115, USA

**Keywords:** cyclin D, cyclin-dependent kinase 4, cell migration, cytoskeletal reorganization, lamellipodia formation

## Abstract

Beyond their canonical role in promoting G1/S progression, the complexes formed by cyclin D and cyclin-dependent kinase (CDK) 4/6 have emerged as contributors to enhanced cell migration. However, a direct link between this complex and cytoskeletal remodeling during cell motility has remained poorly understood. Here, we show that CDK4/6 inhibition in HeLa cells disrupts lamellipodia formation and subsequent focal adhesion assembly, leading to a reduction in cell migration and invasion. Notably, CDK4, but not CDK6, in complex with cyclin D1/D2, localizes to membrane ruffles to facilitate cytoskeletal reorganization. Mechanistically, proteomic and phosphoproteomic analyses revealed that CDK4 inhibition attenuates the transforming growth factor β (TGFβ) pathway via reduced Smad3 phosphorylation at Thr8, downregulating integrin subunits (α5, α6, and β1). Furthermore, CDK4 inhibition significantly decreased focal adhesion kinase (FAK) phosphorylation at Tyr397 and Rac1-GTP levels. Importantly, the resulting migration defect was largely restored by activation of either Rac1 or FAK. Thus, our data support a model in which cyclin D1/D2–CDK4 promotes phosphorylation of Smad3, leading to upregulation of integrin subunits, activation of FAK and Rac1, and consequent lamellipodia formation and cell migration. These findings provide direct evidence that CDK4 regulates actin cytoskeletal reorganization during cell migration and suggest that CDK4/6 inhibitors may dampen cytoskeleton-dependent tumor invasion, in addition to their antiproliferative effects.

## 1. Introduction

Cyclin-dependent kinase (CDK) 4 and 6, in complex with their regulatory subunits cyclin D (D1, D2, or D3), are well-established drivers of the G1/S transition during the cell cycle [1]. Nuclear accumulation of this complex enables the phosphorylation of the retinoblastoma protein (RB), thereby liberating the E2F transcription factor and facilitating the expression of genes critical for DNA replication [2].

Beyond this cell cycle control, growing evidence indicates that the functions of cyclin D–CDK4/6 extend to critical biological processes such as cell migration. For instance, studies using cyclin D1-deficient models have demonstrated that cyclin D1 is essential for the migration of various normal cell types, such as mouse embryonic fibroblasts (MEFs) [3,4], human macrophages [5], and human aortic smooth muscle cells [6]. Furthermore, the cyclin D–CDK4/6 complex has been shown to contribute to migration, invasion, and even metastasis in multiple cancer models, including breast cancer [7], melanoma [8], bladder cancer [9], osteosarcoma [10], colorectal cancer [11], and cervical carcinoma [11]. These findings collectively underscore an indispensable role for cyclin D–CDK4/6 in mammalian cell migration.

Although the role of cyclin D–CDK4/6 in cell migration has been well established, the molecular mechanisms linking this complex to cytoskeletal remodeling remain poorly understood. Cell migration relies on a highly dynamic and polarized cytoskeleton system [12,13]. It is well recognized that the dynamic network of actin filaments and their interplay with actin-binding proteins (ABPs) are critical determinants of cell migration [14]. Upon initiation of migration, extracellular signals trigger cell polarization. At the leading edge, actin polymerizes to extend lamellipodia, broad sheet-like protrusions, and filopodia, slender finger-like extensions. At the advancing front of lamellipodia, cells form focal adhesions (FAs) that connect the extracellular matrix (ECM) to the actin cytoskeleton, thereby anchoring the protrusions and pulling the cell body forward. Finally, to propel forward movement, the cell rear retracts through actomyosin stress fiber contraction and the disassembly of adhesion complexes [15]. A limited number of studies suggest that the cyclin D–CDK4/6 complex may regulate migration by interacting with certain cytoskeleton-associated proteins, such as the ABP filamin A (FLNA) [16], the actin cytoskeleton regulator myristoylated alanine-rich-C-kinase substrate (MARCKS) [17], and the integrin-inactivating protein SHANK-associated RH domain-interacting protein (SHARPIN) [18], or through interactions with epithelial–mesenchymal transition (EMT)-related factors, such as Snail1 and zinc-finger E-box binding homeobox 1 (ZEB1) [7,19]. Nevertheless, direct evidence is still lacking regarding how cyclin D–CDK4/6 regulates cytoskeletal dynamics and the resulting morphological changes that drive cell migration, and the mechanisms underlying these processes remain unclear.

In this study, we show that inhibition of CDK4/6 impairs lamellipodia formation and focal adhesion assembly, reducing cell migration and invasion. We identified a distinct localization of CDK4, but not CDK6, in complex with cyclin D1 or D2 at membrane ruffles. We further demonstrate that cyclin D1/D2–CDK4 activates the focal adhesion kinase (FAK) /Rac1 signaling pathway, inducing lamellipodia formation and promoting focal adhesion assembly, thereby enhancing cell motility. Together, our findings propose a novel mechanism by which cyclin D1/D2–CDK4 regulates actin cytoskeletal remodeling to facilitate migration, providing direct evidence that expands the understanding of the non-canonical functions of CDK4.

## 2. Results

### 2.1. CDK4/6 Inhibition Impairs Cell Migration and Invasion

**Figure 1:**
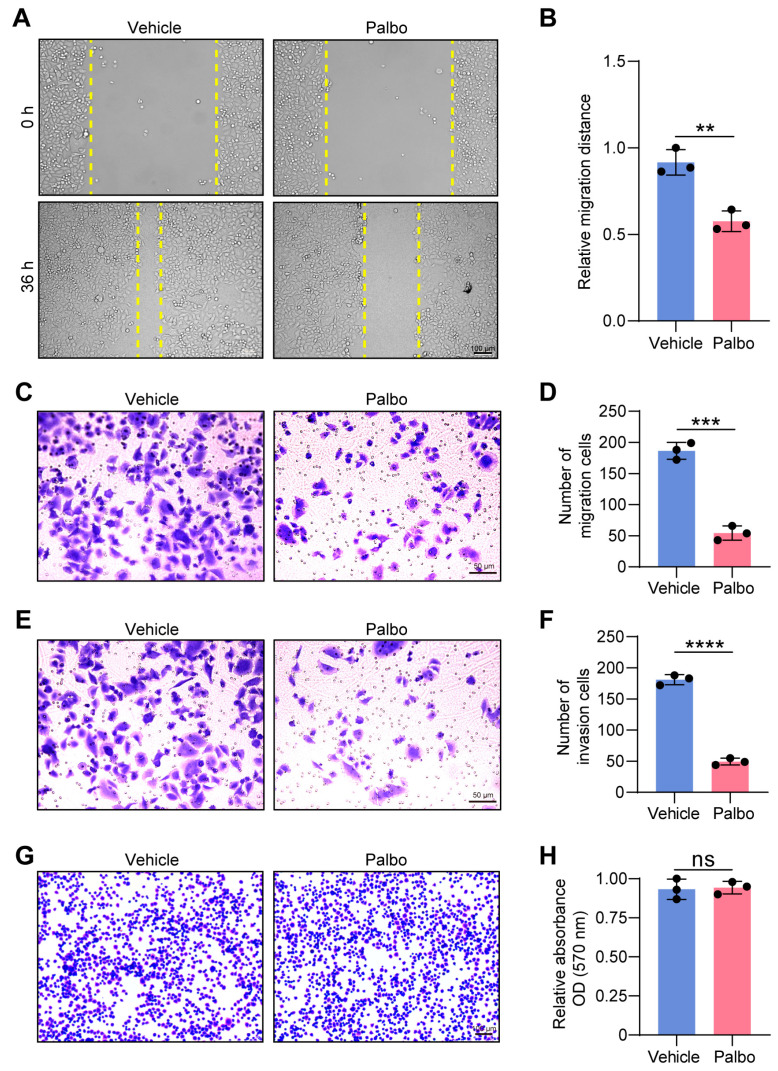
Effect of CDK4/6 inhibition on cell motility. (**A**,**B**) Migration capacity of HeLa cells treated with vehicle or palbociclib (Palbo) was tested by the wound healing assay. Scale bar, 100 μm. The histogram shows the relative migration distance compared to the initial wound at 0 h, measured 36 h after wounding. ** *p* < 0.01. (**C**,**D**) Representative images from the transwell migration assay of HeLa cells treated with vehicle or Palbo over 24 h. Scale bar, 50 μm (**C**). Quantification analysis shows the number of migrated cells (**D**). *** *p* < 0.001. (**E**,**F**) Representative images of transwell invasion assay in vehicle- and Palbo-treated HeLa cells over 24 h. Scale bar, 50 μm (**E**). Quantification analysis shows the number of invading cells (**F**). **** *p* < 0.0001. (**G**,**H**) Analysis of cell adhesion capacity to gelatin of vehicle- and Palbo-treated HeLa cells. Representative images of crystal violet staining showing cells adhered to gelatin-coated plates after 2 h (**G**). Scale bar, 100 μm. Relative adhesion capacity was quantified by measuring the absorbance at 570 nm (OD570), with the vehicle group normalized to 1 (**H**). ns: not significant. All data represent mean ± s.d. from three independent experiments. *p*-value via two-tailed unpaired Student’s *t*-test.

Based on the observation that loss of cyclin D1 inhibits cell migration [11], we asked whether pharmacological inhibition of CDK4/6 activity similarly impairs cell motility. To address this, we used HeLa cells, a well-established model system in basic research, and treated them with 1 μM palbociclib (Palbo), a selective CDK4/6 inhibitor (CDK4/6i), or vehicle control for 48 h. Cell migration was assessed using a wound-healing assay. Quantitative analysis revealed that at 36 h, the wound closure rate in the Palbo-treated group was only 63% of that in the vehicle control, indicating that CDK4/6 inhibition significantly suppressed HeLa cell migration (Figure 1A,B). Consistent results were obtained in a transwell migration assay (Figure 1C,D). We further examined cell invasion and found that CDK4/6i treatment markedly reduced HeLa cell invasiveness by 73% compared to control (Figure 1E,F). Given that cell migration and invasion are dynamic processes dependent on the coordinated regulation of cell adhesion and motility [20], we next assessed whether CDK4/6 influences cell adhesion capacity. However, the cell adhesion assay demonstrated no significant difference in adherent ability between the Palbo- and vehicle-treated groups (Figure 1G,H). Together, these results indicate that CDK4/6 enhances HeLa cell motility by promoting migration and invasion, without substantially affecting cell adhesion.

### 2.2. CDK4/6 Inhibition Disrupts Actin Cytoskeletal Reorganization

**Figure 2:**
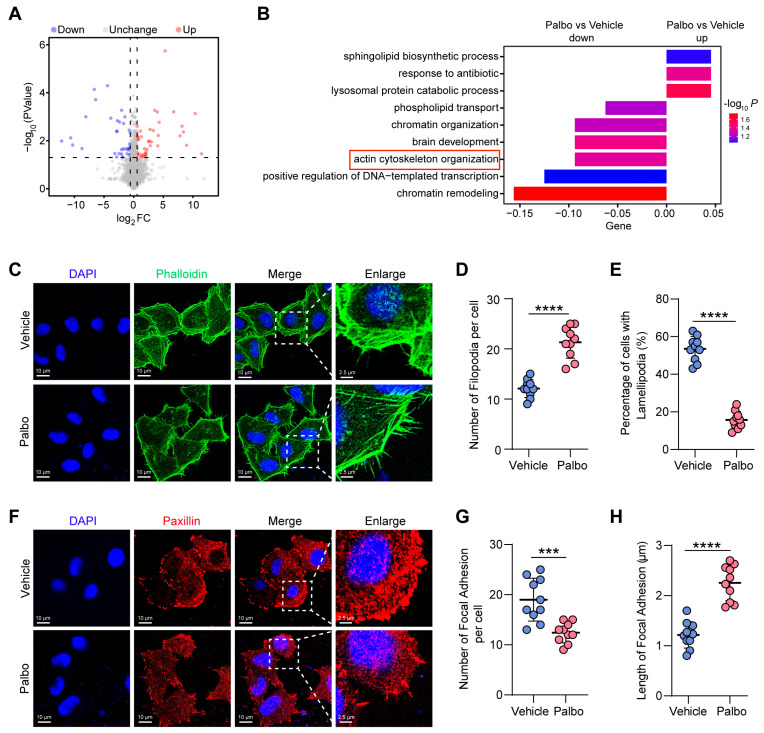
CDK4/6 inhibition impairs actin cytoskeletal remodeling and focal adhesion assembly. (**A**) Volcano plot of differentially expressed proteins in Palbo-treated versus control HeLa cells. (**B**) GO enrichment analysis for up- and downregulated proteins in Palbo-treated HeLa cells compared to controls, false discovery rate (FDR) < 0.01. (**C**) Representative immunofluorescence images of actin cytoskeleton in vehicle- and Palbo-treated HeLa cells, stained with FITC-phalloidin (F-actin, green) and DAPI (nuclei, blue). Scale bars, 10 μm. Enlarged scale bars, 2.5 μm. (**D**) Quantification of filopodia number in HeLa cells treated with vehicle and Palbo (n = 10 cells per condition). **** *p* < 0.0001. (**E**) Percentages of cells with lamellipodia in control and Palbo-treated groups (n = 50 cells per condition). **** *p* < 0.0001. (**F**) Representative immunofluorescence images of focal adhesions in vehicle- and Palbo-treated cells, labeled with paxillin (focal adhesion, red) and DAPI. Scale bars, 10 μm. Enlarged scale bars, 2.5 μm. (**G**,**H**) Quantification of focal adhesion number (**G**) and length (**H**) in vehicle- and Palbo-treated cells (n = 10 per condition). *** *p* < 0.001, **** *p* < 0.0001. All data represent mean ± s.d. from three independent experiments. *p*-value via two-tailed unpaired Student’s *t*-test.

To explore the mechanisms through which CDK4/6 regulates cell migration and invasion, we performed a proteomic analysis comparing CDK4/6i-treated and control HeLa cells. This revealed 78 differentially expressed proteins (DEPs), including 44 upregulated and 34 downregulated proteins (Figure 2A). Gene Ontology (GO) analysis of these downregulated proteins indicated significant enrichment in the actin cytoskeleton organization pathway (Figure 2B). Since actin cytoskeleton organization and turnover are crucial for cell migration [21], we hypothesized that CDK4/6 promotes migration by positively regulating actin cytoskeleton remodeling. To test this, we used fluorescence-conjugated phalloidin to stain actin filaments in control and CDK4/6i-treated cells. We observed a clear morphological shift following CDK4/6 inhibition. While 53.5% of control cells displayed extended lamellipodia, treatment reduced this population to 15.7% and significantly increased the number of filopodia (Figure 2C–E). Lamellipodia are recognized as the primary drivers of cell migration, whereas filopodia function as environmental sensors [22]. Critically, filopodia can act as precursors that initiate the formation and directional extension of lamellipodia, transforming into lamellipodia, and thereby contributing to cell migration [23]. Given this established role, our findings suggest that CDK4/6 inhibition disrupts the actin cytoskeletal reorganization required for this process. Consequently, the filopodia-to-lamellipodia transition is suppressed, thereby impairing lamellipodia formation.

FAs serve as essential structural links between the actin cytoskeleton and the ECM, facilitating cell migration [24]. To determine whether CDK4/6 also influences FA assembly, we performed immunofluorescence staining for paxillin, a well-known FA marker. The results showed that paxillin-positive FAs were abundant and well-formed in control cells, but became fewer and more slender upon CDK4/6 inhibition (Figure 2F–H). This phenomenon indicates that CDK4/6 activity is essential for FA assembly, thereby enabling the transduction of ECM signals to regulate the actin cytoskeleton.

Collectively, these findings demonstrate that CDK4/6 supports cell migration and invasion by mediating actin cytoskeletal reorganization, thereby promoting lamellipodia formation and focal adhesion assembly.

### 2.3. CDK4/6 Inhibition Reduces GTPase Activities of Rac1 and Cdc42

**Figure 3:**
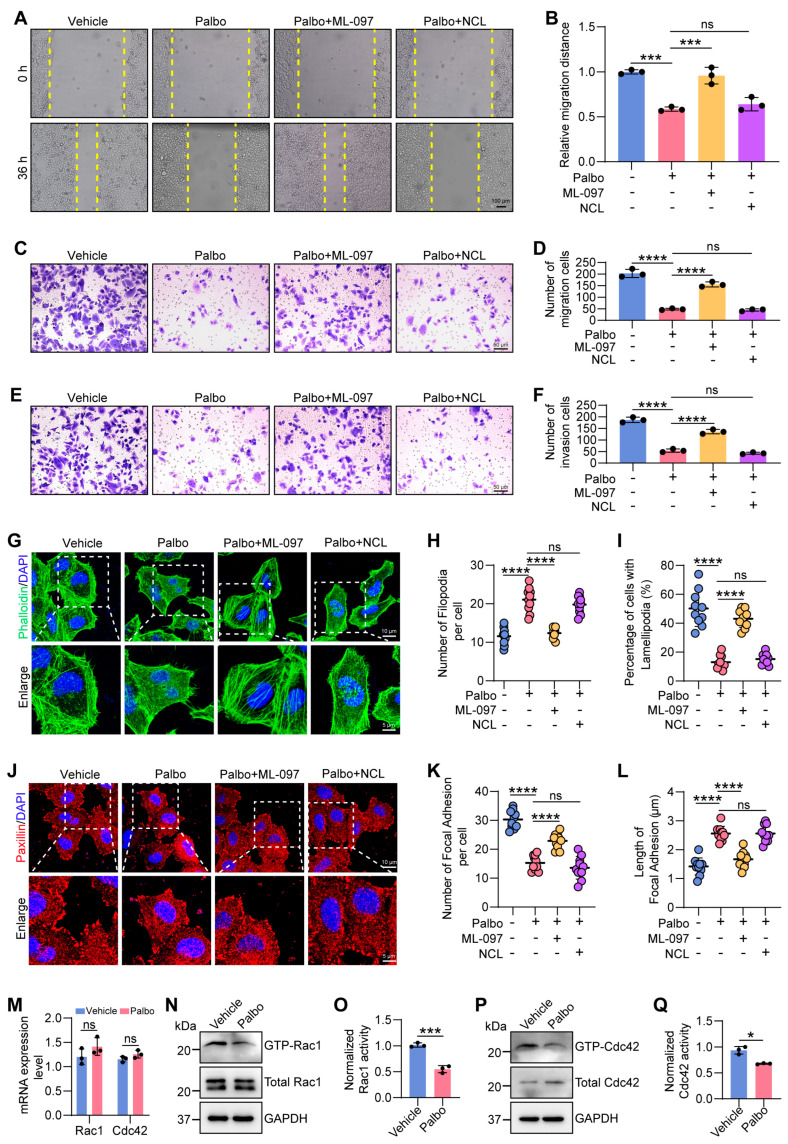
Rac1/Cdc42 activator restores CDK4/6 inhibition-impaired cytoskeletal reorganization and cell migration. (**A**,**B**) Representative images of wound healing scratch assay in vehicle-, Palbo-, Palbo/ML-097, Palbo/Narciclasine (NCL)-treated HeLa cells. Scale bar, 100 μm (**A**). Quantification of relative migration distance at 36 h compared to the initial wound at 0 h (**B**). *** *p* < 0.001. ns: not significant. (**C**,**D**) Transwell migration assay of the four treatment groups after 24 h. Scale bar, 50 μm. **** *p* < 0.0001. ns: not significant. (**E**,**F**) Transwell invasion assay of the four treatment groups after 24 h. Scale bar, 50 μm. **** *p* < 0.0001. ns: not significant. (**G**) Immunofluorescence images of actin cytoskeleton in vehicle-, Palbo-, Palbo/ML-097-, Palbo/NCL-treated HeLa cells. Scale bar, 10 μm. Enlarged scale bar, 5 μm. (**H**) Quantification of filopodia number in the above four treatment groups (n = 10 cells per condition). **** *p* < 0.0001. ns: not significant. (**I**) Percentage of cells with lamellipodia in the four groups (n = 50 cells per condition). **** *p* < 0.0001. ns: not significant. (**J**) Representative immunofluorescence images of focal adhesions in vehicle-, Palbo-, Palbo/ML-097-, Palbo/NCL-treated HeLa cells. Scale bar, 10 μm. Enlarged scale bar, 5 μm. (**K**,**L**) Quantification of focal adhesion number (**K**) and length (**L**) in the four treatment groups (n = 10 cells per condition). **** *p* < 0.0001. ns: not significant. (**M**) qRT-PCR assessing the relative mRNA expression of Rac1 and Cdc42 in vehicle- and Palbo-treated cells. ns: not significant. (**N**–**Q**) Representative Western blot image (**N**,**P**) and quantification (**O**,**Q**) of active GTP-Rac1 and GTP-Cdc42 in vehicle- and Palbo-treated HeLa cells. * *p* < 0.05, *** *p* < 0.001. All data represent mean ± s.d. from three independent experiments. *p*-value via two-tailed unpaired Student’s *t*-test (**O**,**Q**), one-way ANOVA (**B**,**D**,**F**,**H**,**I**,**K**,**L**), or two-way ANOVA (**M**).

Small GTPases from the Rho family are key players in the remodeling of the actin cytoskeleton [25]. We therefore hypothesized that CDK4/6 promotes cytoskeletal organization by activating Rho GTPase signaling. There are three best-characterized Rho GTPase members, Rac1, Cdc42, and RhoA [26]. Accordingly, we performed rescue experiments through co-treating HeLa cells with Palbo and either the Rac1/Cdc42 activator ML-097 or the RhoA activator narciclasine (NCL) to determine whether CDK4/6-mediated cytoskeletal architecture formation is dependent on Rho-family GTPase activation, and which one is essential for this biological process. Wound healing and transwell assays consistently showed that ML-097, but not NCL, largely restored cell migration and invasion impaired by CDK4/6 inhibition (Figure 3A–F). Furthermore, phalloidin staining results showed that actin morphology in cells co-treated with Palbo and ML-097 closely resembled that of control cells, with a higher proportion of cells with lamellipodia and fewer filopodia per cell. In contrast, the Palbo-NCL group exhibited a phenotype similar to Palbo treatment alone, characterized by abundant filopodia and reduced lamellipodia formation (Figure 3G–I). Similarly, ML-097 also partially restored the abundant and robust morphology of FAs, whereas NCL failed to reverse the slender and reduced FAs caused by CDK4/6 inhibition (Figure 3J–L). Together, these results indicate that CDK4/6 promotes actin-dependent migration and invasion primarily through activating Rac1 and Cdc42, which is consistent with the observed impaired filopodia-to-lamellipodia transition upon CDK4/6 inhibition (Figure 2C–E).

Functioning as molecular switches, the Rho GTPases Rac1 and Cdc42 transition between a GTP-bound active state and a GDP-bound inactive state [27]. To further explore how CDK4/6 activates Rac1 and Cdc42, we assessed their mRNA and protein levels, as well as their GTPase activity, following CDK4/6 inhibition. Neither Rac1 nor Cdc42 showed changes in mRNA or total protein levels upon CDK4/6i treatment (Figure 3M,N,P). Notably, the levels of GTP-bound Rac1/Cdc42 were significantly reduced in Palbo-treated cells (Figure 3N–Q), indicating that CDK4/6 activates Rac1 and Cdc42 post-translationally by enhancing their GTPase activity.

In summary, our findings demonstrate that CDK4/6 drives lamellipodia formation and subsequent focal adhesion assembly by specifically increasing the GTPase activity of Rac1, thereby facilitating HeLa cell migration and invasion.

### 2.4. CDK4 Co-Localizes with Cyclin D1/D2 at Lamellipodia

**Figure 4:**
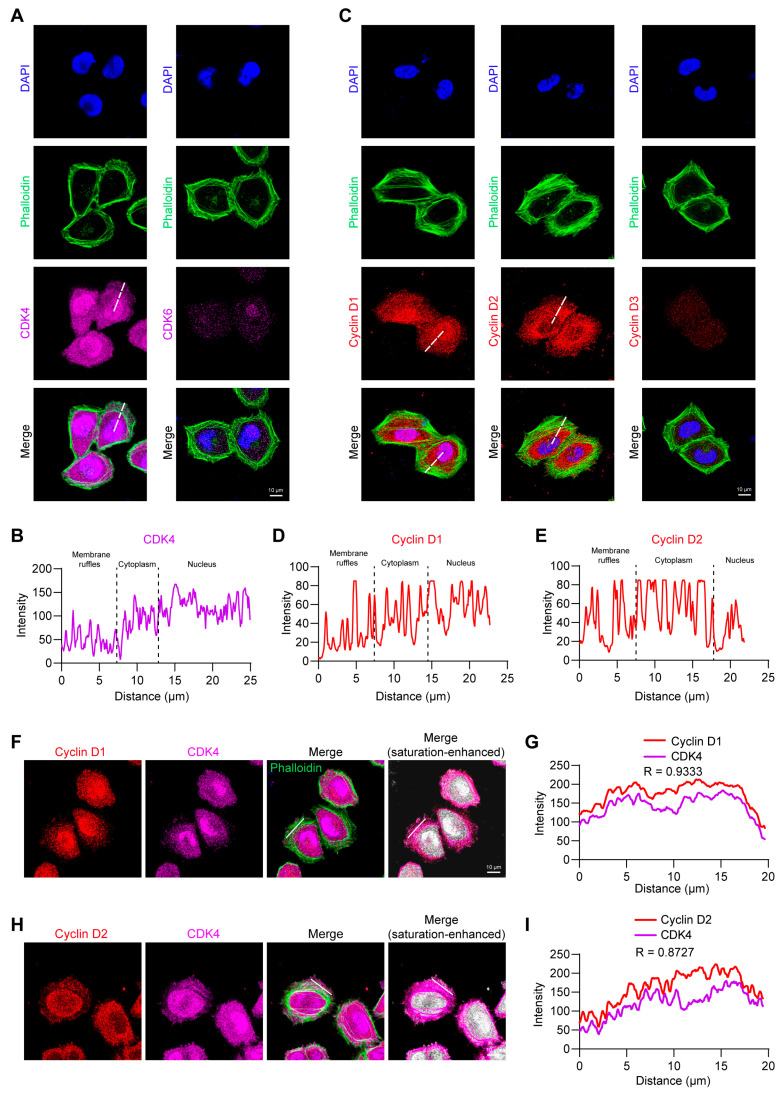
Co-localization of CDK4 with cyclin D1/D2 at the lamellipodia region. (**A**) Subcellular localization of CDK4 and CDK6 in wild-type HeLa cells. Cells were stained for F-actin (FITC-phalloidin, green), CDK4 or CDK6 (purple), and nuclei (DAPI, blue). Scale bar, 10 μm. (**B**) The line-scan profile shows the fluorescence intensity across the membrane ruffles, cytoplasm, and nucleus along the line drawn on the CDK4 image in panel (**A**). (**C**) Subcellular localization of cyclin D1, D2, and D3 (red) in wild-type HeLa cells. Scale bar, 10 μm. (**D**,**E**) The line-scan profile shows the fluorescence intensity across the membrane ruffles, cytoplasm, and nucleus along the line drawn on the cyclin D1/D2 images in panel (**C**). (**F**) Immunofluorescence staining showing co-localization of CDK4 (purple) and cyclin D1 (red) at the lamellipodia region. ‘Merge’ indicates the original merged image; ‘Merge (saturation-enhanced)’ shows the merged image after saturation adjustment to better visualize the co-localization of CDK4 with cyclin D1 within lamellipodia. Scale bar, 10 μm. (**G**) The linear intensity profile along the line shown in the Merge image of panel (**F**). Pearson’s correlation coefficient (PCC) R = 0.9333. (**H**) Immunofluorescence staining showing co-localization of CDK4 (purple) and cyclin D2 (red) at the lamellipodia region. Scale bar, 10 μm. (**I**) The intensity profile along the line shown in the Merge image of panel (**H**). PCC R = 0.8727.

Given that Palbo inhibits the kinase activity of both CDK4 and CDK6, we next aimed to identify which kinase plays the predominant role in cytoskeletal regulation and cell migration. Using immunofluorescence and confocal microscopy, we examined the expression levels and subcellular localization of CDK4 and CDK6 in wild-type HeLa cells. Notably, CDK4 was highly expressed in the nucleus and cytoplasm, and was also present at membrane ruffles (Figure 4A,B). In contrast, CDK6 expression was low in the nucleus and cytoplasm and nearly undetectable in membrane ruffles (Figure 4A). We also assessed the subcellular localization of the regulatory subunits of CDK4/6, three types of cyclin Ds (D1, D2, D3). Cyclin D1 primarily accumulated in the nucleus, but significant distribution signals were also observed in the cytoplasm and membrane ruffles (Figure 4C,D). Interestingly, cyclin D2 signals were predominantly localized in the cytoplasm with negligible nuclear expression, while lower yet detectable signals were present in membrane ruffles (Figure 4C,E). Cyclin D3, similar to CDK6, exhibited very weak expression throughout the cell and was nearly undetectable at the membrane ruffles (Figure 4C). These findings indicate that CDK4, cyclin D1, and cyclin D2 are the principal components involved in actin cytoskeleton-dependent migration in HeLa cells.

We next asked whether CDK4 is directly bound and activated by cyclin D1 or D2 at membrane ruffles, similar to the classical paradigm of cyclin D–CDK4 regulating cell proliferation in the nucleus, and whether this colocalization represents a functional interaction associated with actin cytoskeleton-dependent migration. Double immunofluorescence labeling revealed clear co-localization of CDK4 with both cyclin D1 and cyclin D2 in lamellipodial regions (Figure 4F–I), suggesting that these complexes participate in local cytoskeletal regulation.

### 2.5. CDK4 Upregulates Integrin Expression via the TGFβ/Smad3 Pathway

**Figure 5:**
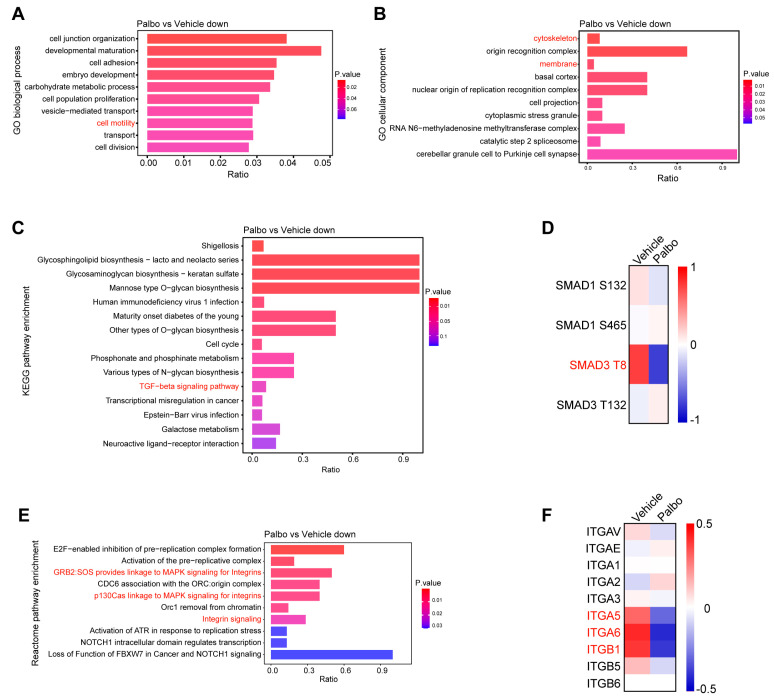
CDK4/6 inhibition downregulates integrin subunits α5, α6, and β1 via TGFβ/Smad3 signaling. (**A**,**B**) Functional enrichment analysis of downregulated phosphopeptides in Palbo-treated versus control HeLa cells, showing biological processes (**A**) and cellular components (**B**). FDR < 0.01. (**C**) KEGG pathway analysis of downregulated phosphopeptides in Palbo-treated versus control cells. FDR < 0.01. (**D**) Heatmap generated from proteomic and phosphoproteomic mass spectrometry data shows the differential phosphorylation of SMAD1 and SMAD3 in vehicle- and Palbo-treated cells. Phosphorylation levels (phosphorylated/total protein) were normalized and are presented as row-wise Z-scores (color scale: −1 to 1). (**E**) Reactome pathway enrichment analysis of downregulated phosphopeptides in Palbo-treated versus control cells. FDR < 0.01. (**F**) Heatmap generated from proteomic mass spectrometry data shows the differentially expressed integrin subunits in vehicle- and Palbo-treated cells. The color scale represents row-wise Z-scores of protein abundance, with values in the range of −0.5 to 0.5.

To elucidate the molecular mechanisms by which CDK4 regulates cytoskeleton-dependent migration in HeLa cells, we performed a phosphoproteomic analysis comparing control and CDK4/6i-treated cells. GO analysis of the downregulated phosphoproteins following CDK4/6 inhibition revealed significant enrichment in terms related to cell motility, with many of these proteins localized to the cytoskeleton and membrane (Figure 5A,B). KEGG pathway analysis further indicated that transforming growth factor β (TGFβ) signaling was markedly downregulated upon CDK4 inhibition, most notably a pronounced reduction in the phosphorylation of its core factor, Smad3, at Thr8 (Figure 5C,D). Previous studies have shown that CDK4 directly phosphorylates Smad3 at multiple residues in vivo, including Thr8, Thr179, and Ser213 [28], supporting the plausibility of CDK4 activating TGFβ signaling via Smad3 T8 phosphorylation.

Reactome pathway enrichment analysis of the downregulated phosphoproteins revealed that three of the top ten enriched pathways were integrin-related signaling (Figure 5E). Since integrin expression, activation, and subsequent binding to the ECM are known to initiate downstream signaling cascades that regulate cytoskeleton dynamics and cell migration [29], we reasoned that integrin signaling might be impaired upon CDK4 inhibition. Moreover, TGFβ/Smad signaling has been reported to positively regulate the expression of integrin subunits such as α6 and β1 [30]. Consistent with this, our proteomic data demonstrated that integrin α5, α6, and β1 subunits (ITGA5, ITGA6, ITGB1) were significantly downregulated in CDK4/6i-treated cells, whereas other subtypes remained largely unchanged (Figure 5F). These findings suggest that CDK4 promotes the expression of integrin subunits α5, α6, and β1 through the TGFβ/Smad3 signaling axis.

In summary, we propose that CDK4 facilitates cytoskeleton-dependent migration by activating the integrin-related pathway via TGFβ/Smad3 signaling. Specifically, CDK4 promotes Smad3 phosphorylation at Thr8, which activates TGFβ signaling and upregulates integrin subunits α5, α6, and β1, thereby triggering the downstream signaling cascades that ultimately drive cell migration.

### 2.6. CDK4 Promotes Cell Migration Through FAK Activation

**Figure 6:**
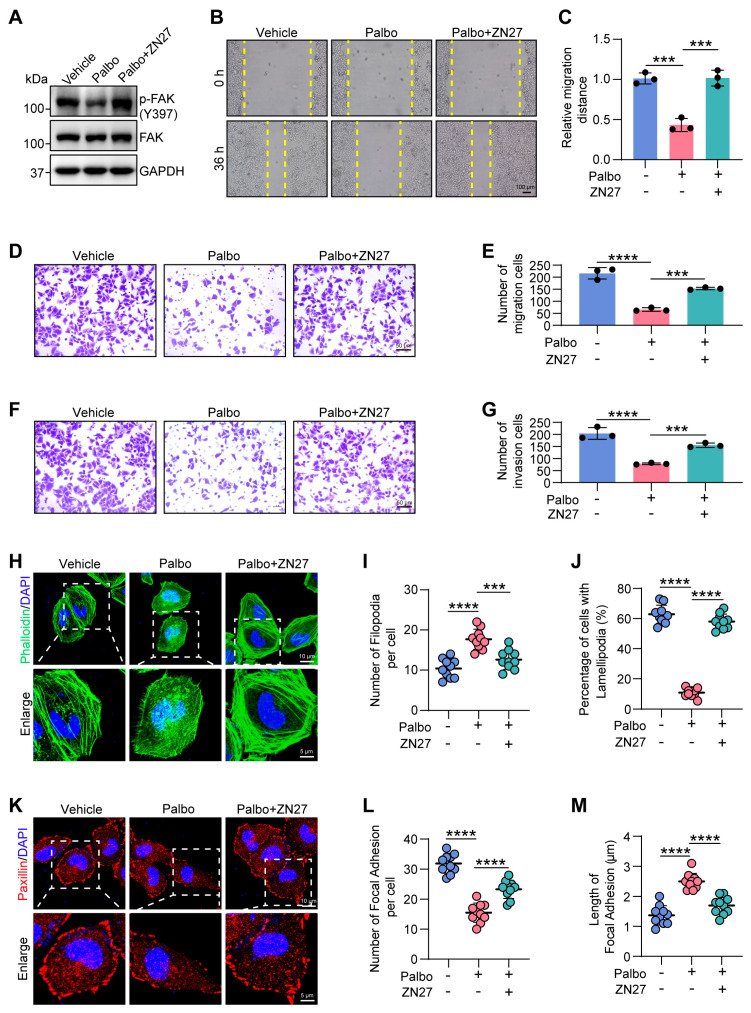
FAK activator restores cytoskeletal reorganization impaired by CDK4/6 inhibition. (**A**) Immunoblotting analysis of phosphorylated FAK (Y397) and total FAK in vehicle-, Palbo-, and Palbo/ZINC40099027 (ZN27)-treated HeLa cells. (**B**,**C**) Wound healing assay in vehicle-, Palbo-, and Palbo/ZN27-treated cells. Scale bar, 100 μm. *** *p* < 0.001. (**D**,**E**) Transwell migration assay of the three treatment groups after 24 h. Scale bar, 50 μm. *** *p* < 0.001, **** *p* < 0.0001. (**F**,**G**) Transwell invasion assay of the three treatment groups after 24 h. Scale bar, 50 μm. *** *p* < 0.001, **** *p* < 0.0001. (**H**) Actin cytoskeleton staining images in vehicle-, Palbo-, and Palbo/ZN27-treated cells. Scale bar, 10 μm. Enlarged scale bar, 5 μm. (**I**) Quantification of filopodia number in the three treatment groups (n = 10 cells per condition). *** *p* < 0.001, **** *p* < 0.0001. (**J**) Percentage of cells with lamellipodia in the three groups (n = 50 cells per condition). **** *p* < 0.0001. (**K**) Staining images of focal adhesion in vehicle-, Palbo-, and Palbo/ZN27-treated cells. Scale bar, 10 μm. Enlarged scale bar, 5 μm. (**L**,**M**) Quantification of the number (**L**) and length (**M**) of focal adhesion in the three treatment groups (n = 10 cells per condition). **** *p* < 0.0001. All data represent mean ± s.d. from three independent experiments. *p*-value via one-way ANOVA.

As a critical oncoprotein, FAK facilitates cell migration by promoting processes such as lamellipodia formation and focal adhesion turnover [31]. Upon binding to the ECM, integrins cluster and become activated, initiating the tyrosine phosphorylation of downstream proteins such as FAK [32]. A key early event in this cascade is the autophosphorylation of FAK at Tyr397, which activates the kinase and promotes downstream signaling that ultimately leads to Rac1 activation [33]. Given the observed downregulation of integrins following CDK4 inhibition, we asked whether this would affect FAK phosphorylation status. In Palbo-treated cells, phosphorylation of FAK at Tyr397 was indeed significantly reduced (Figure 6A), indicating that CDK4 inhibition suppresses FAK activation.

To determine whether FAK acts as a key downstream effector of CDK4 in cytoskeletal reorganization and migration, we asked whether pharmacological activation of FAK could reverse the phenotypic defects caused by CDK4 suppression. We treated cells with the FAK activator ZINC40099027 (ZN27) in combination with Palbo and confirmed that ZN27 enhanced FAK Tyr397 phosphorylation (Figure 6A). Remarkably, FAK activation largely restored cell migration in Palbo-treated cells, as evidenced by both wound healing and transwell migration assays (Figure 6B–E). A similar rescue effect was observed in transwell invasion assays (Figure 6F,G). Furthermore, immunofluorescence analysis showed that ZN27 treatment largely reversed the alterations in actin cytoskeleton organization and focal adhesion dynamics induced by CDK4 inhibition (Figure 6H–M).

Taken together, these results demonstrate that FAK activation is essential for CDK4-promoted cytoskeletal remodeling and cell migration.

## 3. Discussion

In this study, we found that CDK4/6i treatment disrupts actin cytoskeletal reorganization, as evidenced by impaired lamellipodia formation and focal adhesion assembly, ultimately suppressing cell migration and invasion. We further uncovered a novel molecular mechanism governing this process: the cyclin D1/D2–CDK4 complex localizes at the membrane ruffles, activates the TGFβ signaling pathway by promoting the phosphorylation of Smad3 at Thr8, leading to upregulation of integrin subunits α5, α6, and β1. Integrin activation subsequently induces FAK autophosphorylation at Tyr397 and specifically activates Rac1, thereby promoting lamellipodia formation and focal adhesion assembly to facilitate cell migration.

We have shown that CDK4/6 inhibition suppresses HeLa cell migration and invasion—a finding consistent with most existing reports. Since HeLa cells lack functional pRB due to HPV E7 expression [34], this effect can be attributed to a direct regulation of migration, rather than an indirect consequence of proliferative arrest. However, CDK4/6 inhibition does not significantly alter cell adhesion. This functional divergence is further illustrated by studies showing that cyclin D1 deficiency enhances ECM attachment in macrophages and MEFs [2,3], while cyclin D1 overexpression strengthens the adhesion of multiple myeloma cells [35]. These observations suggest that, unlike genetic perturbation of cyclin D1, CDK4/6 inhibition exerts minimal effects on adhesion—particularly in cancer cells exhibiting strong matrix attachment, such as HeLa cells. From the cytoskeleton perspective, previous work has established that cyclin D1 promotes migration of MEFs by suppressing Rho–ROCK signaling, thereby reducing stress fiber-mediated adhesion [4]. Here, we propose that CDK4, in complex with cyclin D1/D2, activates Rac1/Cdc42 via the TGFβ–integrin/FAK pathway, leading to lamellipodia formation and HeLa cell migration. However, the observed increase in filopodia formation appears paradoxical given the concomitant reduction in Cdc42 activity, a GTPase traditionally considered essential for filopodial initiation. We therefore hypothesize that CDK4 may regulate filopodia formation through mechanisms that operate independently of, or in parallel to, canonical Cdc42 signaling. The involvement of cyclin D–CDK4 in Rac1 activation is further supported by a recent study in MEFs, where cyclin D1–CDK4 phosphorylates paxillin to induce membrane ruffling and cell invasion [3]. Notably, we also found that CDK4/6 inhibition impairs FA assembly. Despite the established role of FAs in mediating cell–matrix adhesion, their suppression here did not diminish adhesive capacity. This uncouples FA assembly from the regulation of global adhesion strength under these conditions. We therefore speculate that the observed FA disassembly is likely a downstream effect of the disrupted lamellipodia formation and associated cell morphological changes, which are insufficient to compromise adhesion.

Although cyclin D–CDK4/6 primarily functions in the nucleus to drive G1/S transition [36], our immunofluorescence data reveal significant colocalization of CDK4 with cyclin D1/D2 in the lamellipodia regions, whereas CDK6 and cyclin D3 are barely detectable. This finding strongly implies that cyclin D1/D2–CDK4 complexes at the lamellipodia regions directly modulate cytoskeletal dynamics and cell motility. Supporting this, in breast cancer cells, cyclin D1, though predominantly nuclear, also localizes to the cytoplasm and cell membrane at lower but significant levels [16]. Membrane-localized cyclin D1 colocalizes with Filamin A to drive breast cancer cell migration, and with paxillin in membrane ruffles of MEFs [3,16]. Together, these data indicate that cyclin D1–CDK4 consistently localizes to membranes across diverse cell types, where it regulates cytoskeletal rearrangements. Moreover, we found that cyclin D2, unlike cyclin D1, is predominantly cytoplasmic and also localizes to membrane ruffles, implicating its potential role in cytoskeletal dynamics and even migration. By contrast, the undetectable levels of cyclin D3–CDK6 complexes suggest that this pair may merely function primarily in cell cycle progression.

Mechanistically, our results suggest that CDK4 enhances the phosphorylation of Smad3 at Thr8, which could contribute to activation of TGFβ signaling, thereby enhancing the expression of integrin subunits. As a core component of the TGFβ signaling cascade, Smad3 typically induces G1 cell cycle arrest. Both CDK4 and CDK2 have been shown to phosphorylate Smad3 in vivo and in vitro, thereby inhibiting its activity and suppressing the anti-proliferative effects of TGFβ [37]. Previous studies have identified Thr8, Thr179, and Ser213 as in vivo phosphorylation sites, along with Ser203 and Ser207 as in vitro targets [28]. Among these, phosphorylation at Thr179 is known to be critical for the negative regulation of Smad3 activity. Indeed, CDK2-mediated suppression of Thr179 phosphorylation blocks Pin1-Smad3 interaction and impairs migration in triple-negative breast cancer (TNBC) by altering EMT-related factors [38]. Beyond Thr179, Thr8 phosphorylation has also been shown to be essential for Smad3 activity in cell cycle regulation in T47D cells [39]. Our study now extends the functional significance of Thr8 phosphorylation by linking CDK4-mediated phosphorylation of Smad3 at this site to cell migration, highlighting Thr8 as a potentially critical regulator of Smad3 in the context of motility. The proposed CDK4–TGFβ/Smad3–integrin/FAK axis offers a plausible mechanism for transducing signals from the cell membrane to the cytoskeleton.

Canonically, integrin-mediated FAK activation enhances cyclin D1 transcription and CDK4 activity to promote cell cycle progression [40]. Here, we show the reverse regulatory relationship: CDK4 inhibition reduces the expression of integrin α5, α6, and β1, as well as FAK phosphorylation at Tyr397, and Rac1 activity. Importantly, pharmacological activation of either FAK or Rac1 is sufficient to comparably reverse the actin cytoskeletal disorganization and migration defects induced by CDK4 inhibition, indicating that the FAK-Rac1 axis, which is a well-established pathway [41,42,43], acts as a rate-limiting downstream step in CDK4-mediated cytoskeletal reorganization. Supporting the broader role of CDK family members as upstream regulators of FAK, CDK5 directly phosphorylates FAK at Ser732 to mediate microtubule organization and neuronal migration [44]. Furthermore, our study provides the first evidence linking CDK4 functionally to actin cytoskeletal reorganization and cell migration through the integrin/FAK–Rac1 signaling pathway.

In summary, cyclin D1/D2–CDK4, which extends its known function in cell cycle regulation, modulates cytoskeletal dynamics and promotes cell migration via the TGFβ/Smad3–integrin/FAK–Rac1 axis. This establishes a novel mechanistic link between D-type cyclins/CDKs and the cytoskeletal-migratory machinery in HeLa cells. However, this functional and mechanistic insight needs to be further verified in other experimental models.

## 4. Materials and Methods

### 4.1. Cell Culture

We cultured HeLa cells in Dulbecco’s Modified Eagle’s Medium (Sangon Biotech, Shanghai, China, E600003) augmented with 10% fetal bovine serum (FBS) (Sangon Biotech, Shanghai, China, E510008) and 1% penicillin–streptomycin (Sangon Biotech, Shanghai, China, E607011). The HeLa cell line is routinely tested for mycoplasma contamination and is mycoplasma-free.

### 4.2. Palbociclib and Activator Treatment

To examine the effects of CDK4/6 inhibition and subsequent rescue by pathway-specific activators on lamellipodia formation and cell motility, HeLa cells were first cultured in 0.1% FBS medium and treated for 48 h with the following agents: vehicle control (DMSO), the selective ATP-competitive CDK4/6 inhibitor palbociclib (1 μM) alone [45], or palbociclib in combination with separate activators—ML-097 (50 μM), a pan Ras-related GTPase activator targeting Rac1/Cdc42 [46]; Narciclasine (100 nM), which enhances GTPase RhoA activity [47,48]; or ZINC40099027 (500 nM), a selective activator of FAK that promotes its autophosphorylation at Tyr397 [49]. Following treatment, the medium was replaced with standard 10% FBS growth medium. Cells were then either stimulated for 2 h before lamellipodia analysis or cultured for an extended period to prepare for subsequent migration and invasion assays.

### 4.3. Antibodies and Reagents

Cyclin D1 (Proteintech, Wuhan, China, 26939-1-AP, 1:500 for immunofluorescence (IF)), Cyclin D2 (Proteintech, Wuhan, China, 10934-1-AP, 1:500 for IF), Cyclin D3 (Proteintech, Wuhan, China, 26755-1-AP, 1:500 for IF), CDK4 (Santa Cruz Biotechnology, Dallas, TX, USA, sc-23896, 1:500 for IF), CDK6 (Santa Cruz Biotechnology, Dallas, TX, USA, sc-7961, 1:500 for IF), Phospho-FAK (Tyr397) (Cell Signaling Technology, Danvers, MA, USA, 3283S, 1:1000 for immunoblotting (IB)), FAK (Cell Signaling Technology, Danvers, MA, USA, 3285S, 1:1000 for IB), Paxillin (Abcam, Waltham, MA, USA, ab32084, 1:50 for IF), GAPDH (Proteintech, Wuhan, China, 10494-1-AP, 1:5000 for IB).

Palbociclib hydrochloride (MedChemExpress, Weehawken, NJ, USA, HY-50767A), ML-097 (MedChemExpress, Weehawken, NJ, USA, 743456-83-9), Narciclasine (Aladdin, Shanghai, China, 29477-83-6), ZINC40099027 (MedChemExpress, Weehawken, NJ, USA, HY-134570), Actin-Tracker Green-488 (Beyotime, Shanghai, China, C2201S).

### 4.4. Wound-Healing Assay

HeLa cells were plated at a density of 4 × 10^5^ cells/well in 6-well plates and treated with DMSO, Palbo, or Palbo/activators in a low-serum medium (0.1% FBS) for 48 h. Once the cells formed a confluent monolayer, a straight wound was created in each well using a sterile pipette tip. We captured images of the scars immediately (0 h) and 36 h after scratching using a Leica DMI8 inverted microscope (Leica Microsystems, Wetzlar, Germany). The widths of the wounds at these two time points were measured using ImageJ software (version 1.46). The relative migration distance was calculated as (0 h width minus 36 h width) and normalized to the vehicle control group, which was set to 1.

### 4.5. Migration and Invasion Assay

Following treatment for 48 h, control, palbociclib-treated, and rescue groups (as described above) cells were plated on transwell plates (Corning, Corning, NY, USA, 3422) at a density of 5 × 10^4^ cells/well. Cells were plated in the upper chamber, where the medium had a low serum concentration (0.1% FBS), with the lower chamber containing complete medium to establish a chemotactic gradient. Following a 24-h incubation, cells that had traversed the membrane underwent staining with 1% crystal violet for 30 min. For quantification, the stained cells were imaged and counted using ImageJ software.

### 4.6. Cell Adhesion

Cell adhesion was assessed by the crystal violet stain method. Briefly, following vehicle (DMSO) or palbociclib (1 μM) treatment under normal culture medium for 48 h, cell suspensions (5 × 10^5^ cells/well) were added to gelatin-coated plates. After 1 h of incubation, the adherent cells were subjected to staining with 1% crystal violet (Solarbio, Beijing, China, C8470). The crystal violet attached to the cells was eluted with a 10% acetic acid solution. Finally, we used the optical density of the eluted crystal violet to quantify cell adhesion.

### 4.7. Immunofluorescence

Following 48 h treatments and 2 h stimulation, HeLa cells (vehicle, palbo-treated, and palbo/activator-treated) were fixed, permeabilized, and blocked using 5% normal goat serum (Solarbio, Beijing, China, SL038). After PBS washes, overnight incubation at 4 °C with the indicated antibodies was performed on the samples. The next day, samples were incubated for 1 h with a mixture of corresponding secondary antibodies, Actin-Tracker Green-488 (for F-actin), and DAPI. Stained samples were then mounted on slides and imaged with an ultra-high-resolution Leica Stellaris 5 confocal microscope (Leica Microsystems, Wetzlar, Germany). 

Filopodia and lamellipodia were manually identified based on phalloidin (F-actin) staining and established morphological criteria [50,51]. Filopodia were defined as slender, rod-like protrusions extending from the cell membrane. For quantitative analysis, only protrusions meeting the following criteria were counted as filopodia: a minimum length of 2 µm and clear morphological continuity from the tip to the cell body. Cells displaying flat, sheet-like peripheral extensions were classified as lamellipodia-positive. The percentage of lamellipodia-positive cells was calculated as the number of cells exhibiting lamellipodia divided by the total number of cells assessed, with all counts performed manually.

To quantify the co-localization of cyclin D1 or D2 with CDK4 within the lamellipodial region, Pearson’s correlation coefficient (PCC) analysis was performed. In brief, lamellipodial regions were identified in control HeLa cells based on phalloidin staining (green). A line was drawn at the middle zone between the inner and outer edges of the lamellipodial structure in each of six cells, defining six regions of interest (ROIs). For each ROI, the fluorescence intensity profiles of the cyclin D (red) and CDK4 (purple) channels were obtained via line-scan analysis using ImageJ software. The PCC value was then calculated for each pair of intensity profiles to estimate the degree of co-localization. Data from a representative ROI are presented in the figures.

### 4.8. Quantitative Real-Time Polymerase Chain Reaction (qPCR)

Following isolation with TRIzol reagent (Vazyme Biotech, Nanjing, China, R401-01) from control and palbociclib-treated HeLa cells, total RNA was purified via chloroform/isopropanol precipitation, washed with 70% ethanol, and resuspended. Using the RevertAid First Strand cDNA Synthesis Kit (Thermo Fisher Scientific, Waltham, MA, USA, M16325), RNA was reverse-transcribed into cDNA. Gene expression was then amplified and quantified by qPCR using GoTaq^®^ qPCR Master Mix (Promega, Madison, WI, USA, A6002). The sequences of primers are as follows: Rac1 (5′-CACTGTCCCAACACTCCCAT-3′ and 5′-ACAGCACCAATCTCCTTAGCC-3′), Cdc42 (5′-GTGTGTTGTTGTGGGCGATG-3′ and 5′-TGTGGATAACTCAGCGGTCG-3′).

### 4.9. Rac1 and Cdc42 Kinase Assay

The GTPase activities of Rac1 and Cdc42 were assessed with a Rac1/Cdc42 Activation Assay Combo Kit (Cell Biolabs, San Diego, CA, USA, STA-405). Briefly, protein lysates were prepared from vehicle-treated and palbociclib-treated HeLa cells and equally divided. One portion was used for the measurement of total Rac1/Cdc42, and the other was subjected to pull-down with agarose beads conjugated to the p21-binding domain (PBD) of p21-activated kinase (PAK) to specifically capture the GTP-bound forms. Both total and active Rac1/Cdc42 were finally detected by Western blot analysis.

### 4.10. Western Blotting

Following treatments, vehicle-treated, palbociclib-treated, and palbociclib-plus-activator-treated HeLa cells were lysed in RIPA buffer, supplemented with protease and phosphatase inhibitors. Protein samples (20–30 μg) from the supernatants were resolved via SDS-PAGE and electroblotted onto PVDF membranes. The indicated primary and secondary antibodies were then incubated sequentially with the membranes. Immunoreactive bands were visualized by a Tanon-5200 Chemiluminescence Imaging System (Tanon, Shanghai, China).

### 4.11. Proteomic and Phosphoproteomic Mass Spectrometry Data Analysis

#### 4.11.1. Protein Lysate Preparation

Following a 48-hour low-serum starvation (0.1% FBS) and a 2-hour stimulation with 10% FBS, HeLa cells (control and palbociclib-treated) were trypsinized, collected by centrifugation, and washed with PBS. The cell pellets were then lysed in 8 M urea buffer supplemented with protease inhibitors. Finally, the protein samples were stored at −80 °C.

#### 4.11.2. Protein Digestion

Following reduction with 10 mM TCEP and alkylation with 25 mM CAA (37 °C, 30 min), the protein sample was diluted with 10 mM TEAB. Trypsin was then added at a 50:1 (*w*/*w*) ratio for overnight digestion at 37 °C. The process was stopped the next day by adding formic acid to achieve pH < 3.

For phosphorylated peptide enrichment, phosphorylated peptides were enriched using Fe-NTA magnetic beads. The vacuum-dried peptides were reconstituted in 200 μL of Bind/Wash Buffer (pH < 3.0) and centrifuged. The resulting supernatant was then incubated with pre-washed Fe-NTA beads for 1 h at room temperature with constant shaking at 1000 rpm. The bead-peptide mixture was then loaded onto a C8 StageTip. The beads were washed with 200 μL Bind/Wash Buffer. Subsequently, phosphorylated peptides were eluted by sequential centrifugation with 150 μL of Elution Buffer 1 and 150 μL of Elution Buffer 2 (1000× *g*, 2 min each). The combined eluates were collected and vacuum-dried for subsequent analysis.

#### 4.11.3. LC-MS/MS Analysis

LC-MS/MS analysis was performed using a Q Exactive HF-X mass spectrometer (Thermo Fisher). Following dissolution in 0.1% aqueous formic acid and centrifugation, peptides were separated using a C18 column with an acetonitrile gradient containing 0.1% formic acid. The spray voltage and capillary temperature were set at 2.4 kV and 275 °C, respectively. It acquired full-scan MS spectra (*m*/*z* 350–1500, 120,000 resolution) and data-dependent MS/MS spectra (top 40 ions, 15,000 resolution, 27% HCD collision energy).

For data-independent acquisition (DIA), one full MS scan (*m*/*z* 350–1500) was performed at a resolution of 60,000, followed by 42 MS/MS scans with variable isolation windows (14–312 *m*/*z*) at a resolution of 30,000. The method employed stepped collision energies (25, 27.5, 30%), with the MS2 default charge state set to 3.

#### 4.11.4. Data Analysis

To construct a hybrid spectral library, we processed data-dependent acquisition (DDA) data (6 fractions) and single-shot DIA data to generate individual libraries, which were then merged computationally using Spectronaut v15.7.220308.50606 (Biognosys, Schlieren, Switzerland). The hybrid spectral library served as the reference for protein identification and label-free quantification of the single-shot DIA data. The search utilized the Human UniProt database (retrieved 26 January 2025) with default parameters. Then we applied the Local Normalization algorithm in Spectronaut to normalize the protein intensities. We configured the spectral library generation to require at least three fragments per peptide and to retain a maximum of six most intense fragments. Protein and precursor false discovery rates (FDRs) were controlled at 1%, and protein quantification was reported only for proteins passing the Q-value sparse filtering criterion. Quantitative proteomics data were further processed with R (v4.4.2). Following identification, differentially expressed proteins (adj. *p* < 0.05, |fold change| > 1.5) were plotted using ggplot2. Functional annotations from GO and InterPro were obtained with InterProScan 5. Protein family and pathway enrichments were then analyzed in parallel using the KEGG and Reactome databases, alongside GO enrichment analysis.

### 4.12. Quantification and Statistical Analysis

Statistical analyses were conducted using GraphPad Prism (v8.0.2). Normality of data distribution was analyzed by the Shapiro–Wilk normality test. Comparisons between two groups employed two-tailed Student’s *t*-tests. For analyses involving multiple groups, one-way ANOVA (with Tukey’s test) or two-way ANOVA (with Sidak’s test) was applied as appropriate. A *p*-value < 0.05 was defined as significant, and error bars in all figures represent mean ± standard deviation (s.d.), with specifics provided in the corresponding legends.

## 5. Conclusions

Using the clinically relevant CDK4/6 inhibitor palbociclib, we established a pivotal role for cyclin D1/D2–CDK4 in lamellipodia formation and cell migration and uncovered a novel CDK4–TGFβ/Smad3–integrin/FAK–Rac1 signaling pathway. These findings advance our understanding of the non-canonical functions of CDK4 in cell invasion and metastasis beyond its established role in proliferation, thereby providing a conceptual foundation for more effective use of CDK4/6 inhibitors to suppress tumor invasion and metastasis. A limitation of this study is the reliance solely on CDK4/6 inhibitor-mediated suppression, without validation via alternative approaches like genetic editing. Future work should employ individual knockout of CDK4, CDK6, and cyclins D1-D3 to verify our findings by examining cytoskeletal dynamics and migration/invasion capabilities, thereby clarifying their specific functions within this pathway.

## Data Availability

The original contributions presented in the study are included in the article. Further inquiries can be directed to the corresponding authors. The mass spectrometry proteomics and phosphoproteomics raw datasets presented in this study are openly available in the ProteomeXchange Consortium via the PRIDE [52] partner repository with the dataset identifiers PXD070997 (proteomics) and PXD071048 (phosphoproteomics). The data can be accessed via https://www.ebi.ac.uk/pride/archive/projects/PXD070997 (accessed on 4 December 2025) and https://www.ebi.ac.uk/pride/archive/projects/PXD071048 (accessed on 4 December 2025).

## Abbreviations

The following abbreviations are used in this manuscript:

CDK	Cyclin-Dependent Kinase
TGFβ	Transforming Growth Factor β
FAK	Focal Adhesion Kinase
ABPs	Actin-Binding Proteins
FAs	Focal Adhesions
ECM	Extracellular Matrix
EMT	Epithelial-Mesenchymal Transition
DEPs	Differentially Expressed Proteins
GO	Gene Ontology
MEFs	Mouse Embryonic Fibroblasts
TNBC	Triple-Negative Breast Cancer
FBS	Fetal Bovine Serum
PCC	Pearson’s Correlation Coefficient
ROIs	Regions of Interest
PBD	P21-Binding Domain
PAK	P21-Activated Kinase
DIA	Data-Independent Acquisition
DDA	Data-Dependent Acquisition
FDR	False Discovery Rate
